# Supplementary material for: EZH2-H3K27me3 mediated KRT14 upregulation promotes TNBC peritoneal metastasis
Source: Nat Commun. 2022 Nov 29;13:7344. doi: 10.1038/s41467-022-35059-x (PMC9708848; doi:10.1038/s41467-022-35059-x)
Supplement: Supplementary file 1 — Supplementary Information [file 41467_2022_35059_MOESM1_ESM.pdf]

Supplementary Information

EZH2-H3K27me3 mediated KRT14 upregulation promotes TNBC peritoneal metastasis *By Verma et.al.,*

Division of Cancer Biology, CSIR-Central Drug Research Institute (CDRI), Lucknow, 226031, India

(A)

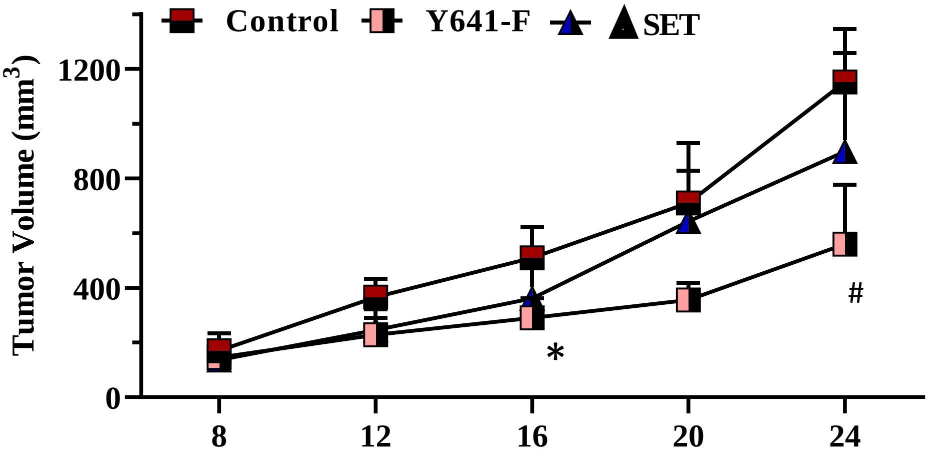

Supplementary Figure: 1

Effect of EZH2 catalytic hyper-activation (Y641-F) and protein overexpression (ΔSET OE) on TNBC tumor growth

The control, EZH2 (Y641-F) OE and Δ SET OE 4T-1 (1X106) cells in 100 µl PBS were subcutaneous inoculated in the left flank of 4- to 6-week-old female nude Crl: CD1-Foxn1nude mice (n=5 each group) and allowed to grow for 25 days (n=4) The growth curve is shown; points are indicative of an average of tumor volume (error bar, +/- SD); * *P*= 0.0365 and #*P*=0.0111, compared to control group, Two Way ANOVA, Dunnett's multiple comparisons test.

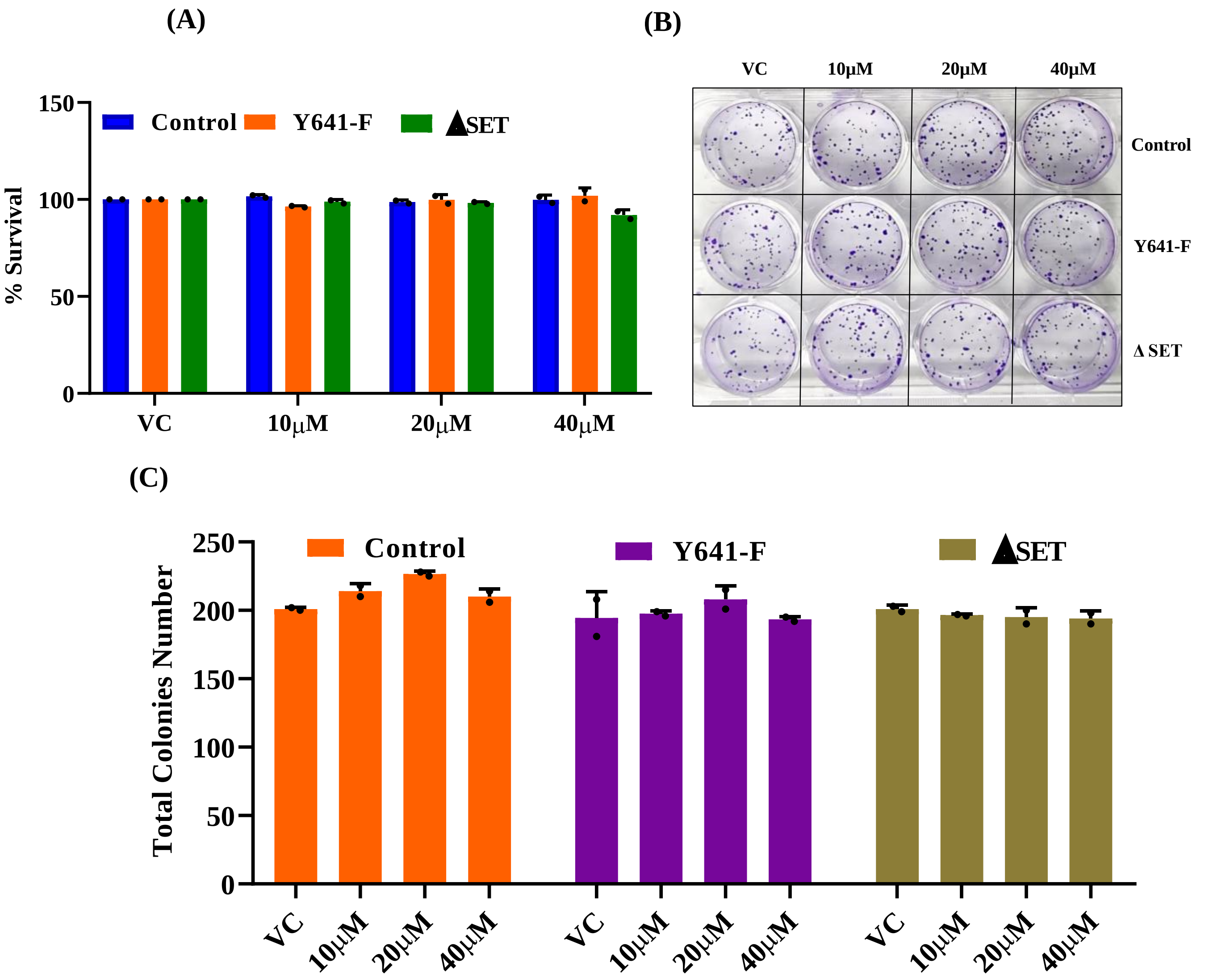

Supplementary Figure: 2

The EPZ6438 treatment has no toxic effect against control, EZH2 Y641-F (OE) and Δ SET (OE) 4T-1 cells

(A) The control, EZH2 Y641-F (OE) and Δ SET (OE) 4T-1 cells were treated with the vehicle and different doses of EPZ6438 (10µM, 20µM and 40 µM) for 48 h and subjected to MTS assay. Data points are mean of duplicate readings of samples; error bars, ± S.D. Two Way ANOVA, Turkey's multiple comparisons test. **(B)** The control, EZH2 Y641-F (OE) and Δ SET (OE) 4T-1 cells were seeded at 200 cells per well in 12 well plate, after 24 h the adhered cells were treated either with vehicle and different doses of EPZ6438 ((10µM, 20µM and 40 µM) and incubated for 2 week at 37^{0}C.The cells were fixed in the crystal violet dye for 1 hr. The picture of stained colonies were taken. **(C)** The stained colonies were counted by Image J Software and represented in the form of bar graph. Data points are mean of duplicate readings of samples; error bars, ± S.D. Two Way ANOVA, Turkey's multiple comparisons test.

(A) (B)

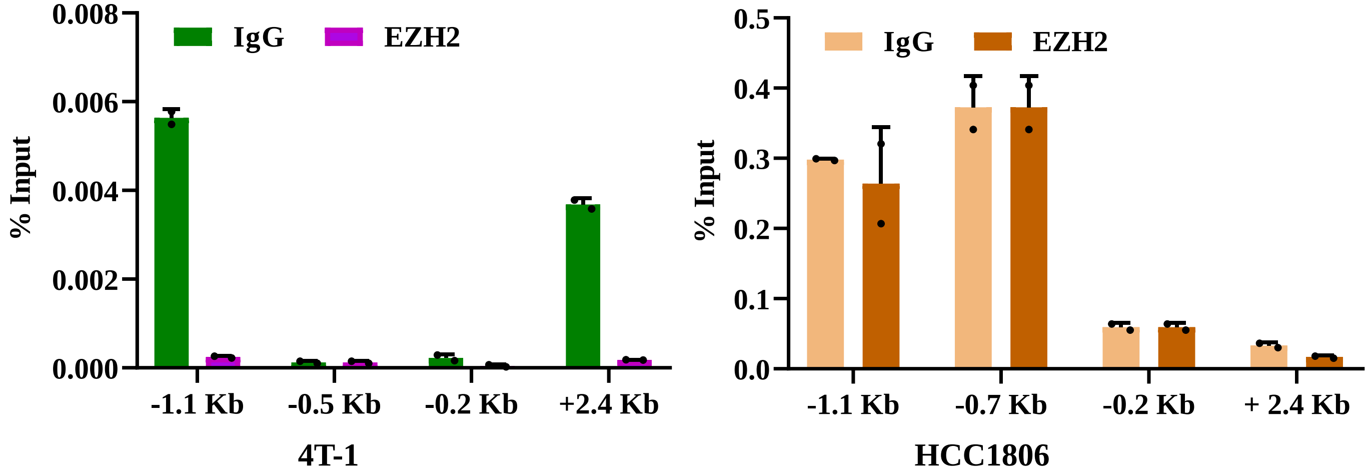

Supplementary Figure: 3

The EZH2 enrichment analysis by ChIP-q PCR at *KRT14* promoter

ChIP was performed in 4T-1 mouse and HCC1806 human using anti-EZH2 and IgG antibodies and then examined by real-time q-PCR. using respective primers of *KRT14* gene. ChIP q-PRC results showing differential fold change in EZH2 enrichment at the promoter of *KRT14* gene. **(A and B)** The analysis of enrichment for EZH2 in the KRT14 Promoter at -0.2 Kb , -0.5 Kb, -1.1 Kb and +2.4 Kb regions from TSS respectively. Data points are mean of duplicate readings of samples; error bars, ± S.D. Two-way ANOVA, Sidak's multiple comparisons test.

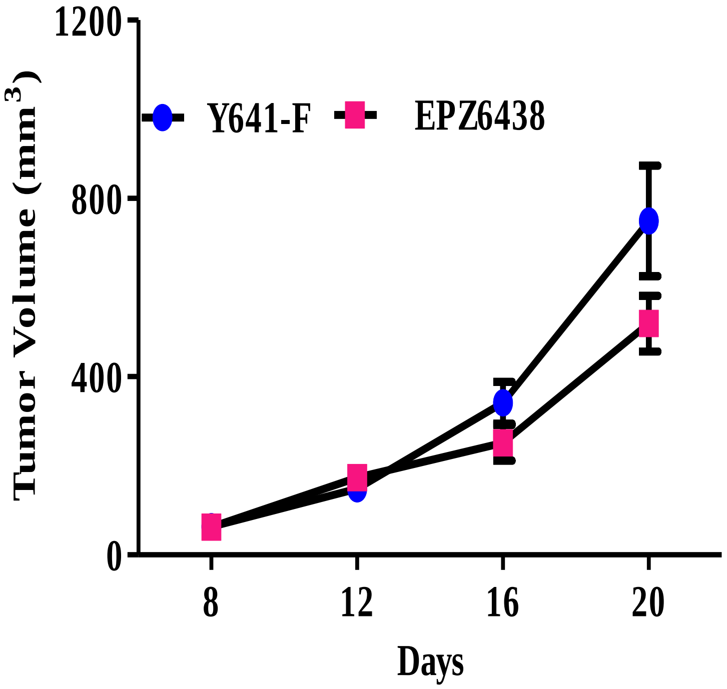

Supplementary Figure: 4

EPZ6438 treatment has no significant impact on tumor growth:

(A) The EZH2 (Y641-F) ($1X10^6$) cells in 100 µl PBS were orthotopically inoculated in the left mammary fat pad of 4- to 6-week-old female nude and allowed to grow for 25 days. After 1 week mice were randomized and divided into vehicle and treatment groups (n=5). The EPZ6438 (250mg/Kg) dose was administered to mice once in a day by oral route. The growth curve analysis of control and EPZ6438 treated mice are shown, data points are indicative of an mean of tumor volume (error bar, +/- SEM). Student's test (Two –sided).

Supplementary Table 1

100 top ranked genes obtained from GSEA analysis were explored in the literature for cancer cell migration and metastasis phenotype and 16 genes were found to be correlated with the aforementioned phenotype and represented in the tabular form with relevant references.

**Gene**	**Reference**
CCNG2	Bernaudo et al., 2016[1], Wang et al.,2020 [2], Yang et al., 2020[3]
NDRG1	Mi et al., 2017 [4]
NCAM1	Gaun et al., 2020 [5]
CBS	Zhang et al., 2022[6]
BNIP3	Chourasia et al., 2015 [7]
ARRB1	Bonstandbad et al., 2021 [8]
AQP1	Ji et al., 2021[9]
KRT16	Huang et al., 2019 [10]
SEMA3C	Tam et al., 2017 [11]
ADAMTS1	Esselens et al., 2010[12]
CCN2	Chin et al., 2011 [13]
CYP1B1	Kwon et al., 2016 [14]
KRT14	Bilandzic et al., 2019[15], Cheung et al., 2016[16]
TM4SF1	Cao et al., 2016 [17]
JAG1	Dia et al., 2014 [18]
NIFK	Lin et al., 2016 [19]

Supplementary Table 2

List of Primers

Quantitative PCR (qPCR) Primers			
**S. No.**	**Gene Name**	**Primer Name (Mouse)**	**Sequence (5'-3')**
1	*CCNG2*	qCCNG2_FP	TTGCACTTGTACCACGCGAT
		qCCNG2_rP	TTGGAGAAGACAACTCGGCA
2	*NDRG1*	q_NDRG1FP	ATGGTAGAGGGTCTCGTGCT
		qNDRG1_RP	CGGGTTCATGTCGTTGAGGA
3	*NCAM-1*	qNCAM1_FP	CGCAGGTGCAGTTTGATGAG
		qNCAM1_RP	CGTACCGAGTACCTCGTCTC
4	*CBS*	qCBS_FP	TCAGGGGACAAGGATCGAGT
		qCBS_RP	TGACCATAGGGGTGTTCCCA
5	*BNIP3*	qBNIP3 _FP	TCACTGTGACAGCCCACCTC
		qBNIP3_RP	ATCTTCCTCAGACAGAGTGCTG
6	*ARRB1*	qARRB1_3 FP	CCAACGTACAGTCGTTCCCA
			CAAGCCTTCCCCGTGTCTTC
7	*AQP1*	qAQP1_FP	ATCCTCCGGGCTGTCATGTA
		qAQP1_RP	AGCTGCAGAGTGCCAATGAT
8	*SEMA3C*	qSEMA3C_FP	GGCGAGGGATCCATACTGTG
		qSEMA3C_RP	TTGAACCCTCGGCATTGTGT
9	*KRT14*	qKRT14_FP	CAGTCCCAGCTCAGCATGAA
		qKRT14_RP	TGGGAAGATGAAAGGTGGGC
10	*ADMTST1*	q ADMST1_FP	CACGTGTGACACTCTCGGAA
		qABMST1_RP	TGGCACAGTGCTTAGCATCA
11	*CYP1B1*	qCYP1B1_FP	AAGTGGCCTAACCCAGAGGA
		qCYP1B1_RP	ATTGCACTGATGAGCGAGGA
12	*KRT16*	qKRT16_FP	TGCGCAAGGTGCTAGATGA
		qKRT16_RP	GACCCCTCAAGGCAAGCATC
13	*TM4SF1*	qTM4SF1_FP	TGCTGCGGCTACGAAAACTA
		qTMFSF1_RP	CATACTCCATGGGCATCGCT
14	*JAG1*	qJAG1_FP	CTCGGGTCAGTTTGAGCTGG

		qJAG1_RP	AGTGACGCGGGACTGATACT
15	*NIFK*	qNIFK_FP	CTGGGCCACCTACCTTCAAC
		qNIFK_RP	TGCTGTTTCCAGTCCGCTTA
16	*CCN2*	qCCN2_FP	AGAACTGTGTACGGAGCGTG
		qCCN2_RP	GTGCACCATCTTTGGCAGTG
17	*SP1*	qSP1_FP	TTCAACAACTACTACCACCAGCA
		qSP1-RP	AGACCCTTGTAGCCCACCAA

**S. No.**	**Gene Name**	**Primer Name (Human)**	**Sequence (5'-3')**
15	*KRT14*	qKRT14_FP	TCTGAACGAGATGCGTGACC
		qKRT14_RP	TGAAGAACCATTCCTCGGCA
16	*SP1*	qSP1_FP	ATACAGGCGAGAGGCCATTT
		qSP1_RP	TGAAGCGCTTAGGACACTCA

Supplementary Table 3

List of ChIP primers.

**Chromatin Immunoprecipitation-Quantitative PCR (ChIP-qPCR) Primers**			
**S. No.**	**Gene Name**	**Primer Name**	**Sequence (5'-3')**
1	*KRT14 (mouse)*	Us_0.2Kb_FP	GGACGAGAAAGCCCAAAACAC
		Us_0.2Kb_RP	CCCGATCAGATCCCTCCTCT
2	*KRT14 (Mouse)*	Us_0.5 Kb_FP	CCAGCTAAGTGCCAGTCTCC
		Us_0.5Kb_RP	AGTAGGGCCTTACCACACCA
3	*KRT14 (Mouse)*	Us_1Kb_FP	GTTCCTCCTCCCCATACGTG
		Us_1Kb_RP	TAAGGGCACATGCCTGGAAC
4	*KRT14 (Mouse)*	Us_1.5Kb_FP	GGAAGGGTCAGGTGGGATTG
		Us_1.5Kb_RP	GAGGCTCCTGCACTGTTCTT
5	*KRT14 (Mouse)*	Ud_2.4Kb_FP	GCCAGCTTAAGAGGGTGGAT
		Ud_2.4 Kb_RP	ACTGATCCCGCATCTCGTTC
6	*KRT14(Human)*	Us_0.2Kb_FP	CGGGACAAGAAAGCCCAAAA
		Us_0.2Kb_RP	TATACTCGTGGGTAGGGGGC
7	*KRT14(Human)*	Us_0.7Kb_FP	GGGTGGGAACCACGATACAC
		Us_0.7Kb_RP	ATGGATACCCGGCTGGAAAG
8	*KRT14(Human)*	Us_1.1Kb_FP	CAGTTCCACAAGGGGCTCAA
		Us_1.1Kb_RP	AGAAGCCTCGTTGGCATTGT
9	*KRT14(Human)*	Us_1.5Kb_FP	TTTGCTGGCAGATTTGGGGA
		Us_1.5Kb_RP	GCCTGACGCATCCTATCTCC
10	*KRT14 (Human)*	Ud_2.4kb_FP	TTCTCTGGGGTCATTCCAGGT
		Ud_2.4Kb_RP	ACTTTTCCATATAGTTCTCACCTCC

Supplementary Table 4

List of ShRNA oligos.

ShRNA Oligos			
**S. No.**	**Gene Name**	**Primer Name**	**Sequence (5'-3')**
1	*KRT14 (Human)*	KRT14_Sh1_FP	CCGGGCCTGCTGAGATCAAAGACTACTCGAGTAGTCTTTGATCTCAGCAGGCTTTTTG
	KRT14 (Human)	KRT14_Sh1_RP	AATTCAAAAAGCCTGCTGAGATCAAAGACTACTCGAGTAGTCTTTGATCTCAGCAGGC
2	*KRT14 (Human)*	KRT14_sh2_FP	CCGGGGTGCAGAGCGGCAAGAGCGACTCGAGTCGCTCTTGCCGCTCTGCACCTTTTTG
		KRT14_sh2_RP	AATTCAAAAAGGTGCAGAGCGGCAAGAGCGACTCGAGTCGCTCTTGCCGCTCTGCACC
3	*KRT14 (Mouse)*	KRT14_Sh1_FP	CCGGCCAATTCTCCTCATCCTCTCACTCGAGTGAGAGGATGAGGAGAATTGG TTTTTG
		KRT14_Sh1_RP	AATTCAAAAACCAATTCTCCTCATCCTCTCACTCGAGTGAGAGGATGAGGAGAATTGG
4	*KRT14 (Mouse)*	KRT14_Sh2_FP	CCGGGCCCACTGAGATCAAAGACTACTCGAGTAGTCTTTGATCTCAGTGGGC TTTTTG
		KRT14_Sh2_RP	AATTCAAAAAGCCCACTGAGATCAAAGACTACTCGAGTAGTCTTTGATCTCAGTGGGC
5	*EZH2 (Mouse)*	EZH2_Sh1_FP	CCGGGCACAAGTCATCCCGTTAAAGCTCGAGCTTTAACGGGATGACTTGTGCTTTTTG
		EZH2_Sh1_RP	AATTCAAAAACACAAGTCATCCCGTTAAAGCTCGAGCTTTAACGGGATGACTTGTGC
6	*SP1 (Human)*	SP1_Sh1_FP	CCGGGCTGGTGGTGATGGAATACATCTCGAGATGTATTCCATCACCACCAGCTTTTTG
		SP1_Sh1_RP	AATTCAAAAAGCTGGTGGTGATGGAATACATCTCGAGATGTATTCCATCACCACCAGC
7	*SP1 (Mouse)*	SP1_Sh1_FP	CCGGCCTTCACAACTCAAGCTATTTCTCGAGAAATAGCTTGAGTTGTGAAGGTTTTTG
		SP1_Sh1_RP	AATTCAAAAACCTTCACAACTCAAGCTATTTCTCGAGAAATAGCTTGAGTTGTGAAGG
8	*UTX (Mouse)*	UTX_Sh1_FP	CCGGCCTTCTCCTAAGTCCACTGAACTCGAGTTCAGTGGACTTAGGAGAAGGTTTTTG
		UTX_Sh1_RP	AATTCAAAAACCTTCTCCTAAGTCCACTGAA**CTCGAG**TTCAGTGGACTTAGGAGAAGG

Supplementary Table 5

Luciferase KRT14 promoter primers:

S.No.	Gene Name	Primer Name	Sequence
1.	*KRT14 (Human)*	KRT14_-1.1 Kb _F	GTAGATCTCTAGGCCTGTTCCTGGATGCAG
		KRT14_-1.1 Kb _R	AGAAGCTTGTAAATTGGAAAGGGATGCGAGTGC
2.	*KRT14 (Human)*	KRT14_-0.5 Kb _F	GGTAGATCTGACCTCCTTGTCTCTAATAGAGGGTCATG
		KRT14_-0.5 Kb _R	ACTAAGCTTGTGAAGAGAAGGTGCTCGGGTAAAT

Supplementary Table 6

TNBC Patient Details

	CASE1	CASE2	CASE3	CASE4	CASE 5	CASE 6	CASE 7	CASE 8	CASE 9	CASE 10
**Diagnosis**	CA Breast	CA Breast	CA Breast	CA Breast	CA Breast	CA Breast	CA Breast	CA Breast	CA Breast	CA Breast
**Consent**	Yes	Yes	Yes	Yes	Yes	Yes	Yes	Yes	Yes	Yes
**Age**	58-62	57-61	58-62	54-58	39-43	69-73	41-45	38-42	71-73	57-61
**Country**	India	India	India	India	India	India	India	India	India	India
**Type**	Human Breast	Human Breast	Human Breast	Human Breast	Human Breast	Human Breast	Human Breast	Human Breast	Human Breast	Human Breast
**Clinical staging**	T2N1M0	T2N1M0	T2N1M0	T3N1M0	T2N0M0	T2N1M0	T2N1M0	T3N1M0	T3N0M0	T2N1M0
**Specimen procedure**	MRM	MRM	MRM	MRM	BCS	MRM	MRM	MRM	MRM	MRM
**Histologic Type**	IDC NST, GRADE III	IDC NST, GRADE III	IDC NST, GRADE III	IDC NST, GRADE III	METAPLASTIC CARCINOMA,GRADE III	IDC NST, GRADE II	IDC NST, GRADE II	IDC NST, GRADE II	IDC NST, GRADE III	IDC NST, GRADE III
**Morphological grade**	3	3	3	3	3	2	3	2	3	3
**Ductal carcinoma in situ**	Absent	Present	Present	Present	Absent	Present	Present	Present	Present	Absent
**LN Status**	Negative	Positive	Negative	Positive	Negative	Positive	Positive	Positive	Positive	Positive

**Pathological staging**	pT2N0	pT2N1a	pT2N0	pT2N3a	pT2snN0	pT2N2a	pT2N2a	pT2N3a	pT2N2a	Pt2n1
**Stage grouping**	IIA	IIB	IIA	IIIC	IIA	IIIA	IIIA	IIIC	IIIA	IIB
**Estrogen receptor status**	Negative	Negative	Negative	Negative	Negative	Negative	Negative	Negative	Negative	Negative
**Progesterone receptor status**	Negative	Negative	Negative	Negative	Negative	Negative	Negative	Negative	Negative	Negative
**HER-2 status**	Negative	Negative	Negative	Negative	Negative	Negative	Negative	Negative	Negative	Negative
**TNBC**	Yes	Yes	Yes	Yes	Yes	Yes	Yes	Yes	Yes	Yes
**Recc/Prog if any,**	Yes	Yes	Yes	Yes	Yes	Yes	Yes	Yes	Yes	Yes
**Metastatic Site**	Lung	Lung	Lung	Breast	Mammary LN	Axillary LN	Pleural Biopsy	Bronchial (CARINA)	Pleural Biopsy	LIVER

References:

1. Bernaudo, S. *et al.* Cyclin G2 inhibits epithelial-to-mesenchymal transition by disrupting Wnt/beta-catenin signaling. *Oncogene* **35**, 4816-27 (2016).
2. Wang, D. *et al.* Cyclin G2 Inhibits Oral Squamous Cell Carcinoma Growth and Metastasis by Binding to IGFBP3 and Regulating the FAK-SRC-STAT Signaling Pathway. *Front Oncol* **10**, 560572 (2020).
3. Yang, J. *et al.* Upregulation of lncRNA LINC00460 Facilitates GC Progression through Epigenetically Silencing CCNG2 by EZH2/LSD1 and Indicates Poor Outcomes. *Mol Ther Nucleic Acids* **19**, 1164-1175 (2020).
4. Mi, L. *et al.* The metastatic suppressor NDRG1 inhibits EMT, migration and invasion through interaction and promotion of caveolin-1 ubiquitylation in human colorectal cancer cells. *Oncogene* **36**, 4323-4335 (2017).
5. Guan, G. *et al.* Upregulation of Neural Cell Adhesion Molecule 1 (NCAM1) by hsa-miR-141-3p Suppresses Ameloblastoma Cell Migration. *Med Sci Monit* **26**, e923491 (2020).
6. Zhang, Y. *et al.* Overexpression of CBS/H2S inhibits proliferation and metastasis of colon cancer cells through downregulation of CD44. *Cancer Cell Int* **22**, 85 (2022).
7. Chourasia, A.H. *et al.* Mitophagy defects arising from BNip3 loss promote mammary tumor progression to metastasis. *EMBO Rep* **16**, 1145-63 (2015).
8. Bostanabad, S.Y., Noyan, S., Dedeoglu, B.G. & Gurdal, H. Overexpression of beta-Arrestins inhibits proliferation and motility in triple negative breast cancer cells. *Sci Rep* **11**, 1539 (2021).
9. Ji, Y., Liao, X., Jiang, Y., Wei, W. & Yang, H. Aquaporin 1 knockdown inhibits triple-negative breast cancer cell proliferation and invasion in vitro and in vivo. *Oncol Lett* **21**, 437 (2021).
10. Huang, W.C. *et al.* A novel miR-365-3p/EHF/keratin 16 axis promotes oral squamous cell carcinoma metastasis, cancer stemness and drug resistance via enhancing beta5-integrin/c-met signaling pathway. *J Exp Clin Cancer Res* **38**, 89 (2019).
11. Tam, K.J. *et al.* Semaphorin 3 C drives epithelial-to-mesenchymal transition, invasiveness, and stem-like characteristics in prostate cells. *Sci Rep* **7**, 11501 (2017).
12. Esselens, C. *et al.* The cleavage of semaphorin 3C induced by ADAMTS1 promotes cell migration. *J Biol Chem* **285**, 2463-73 (2010).
13. Chien, W. *et al.* Expression of connective tissue growth factor (CTGF/CCN2) in breast cancer cells is associated with increased migration and angiogenesis. *Int J Oncol* **38**, 1741-7 (2011).
14. Kwon, Y.J. *et al.* CYP1B1 Enhances Cell Proliferation and Metastasis through Induction of EMT and Activation of Wnt/beta-Catenin Signaling via Sp1 Upregulation. *PLoS One* **11**, e0151598 (2016).
15. Bilandzic, M. *et al.* Keratin-14 (KRT14) Positive Leader Cells Mediate Mesothelial Clearance and Invasion by Ovarian Cancer Cells. *Cancers (Basel)* **11**(2019).
16. Cheung, K.J. *et al.* Polyclonal breast cancer metastases arise from collective dissemination of keratin 14-expressing tumor cell clusters. *Proc Natl Acad Sci U S A* **113**, E854-63 (2016).
17. Cao, J. *et al.* TM4SF1 Regulates Pancreatic Cancer Migration and Invasion In Vitro and In Vivo. *Cell Physiol Biochem* **39**, 740-50 (2016).
18. Dai, Y. *et al.* Silencing of Jagged1 inhibits cell growth and invasion in colorectal cancer. *Cell Death Dis* **5**, e1170 (2014).
19. Lin, T.C. *et al.* The nucleolar protein NIFK promotes cancer progression via CK1alpha/beta-catenin in metastasis and Ki-67-dependent cell proliferation. *Elife* **5**(2016).
