# Supplementary material for: EZH2-H3K27me3 mediated KRT14 upregulation promotes TNBC peritoneal metastasis
Source: Nat Commun. 2022 Nov 29;13:7344. doi: 10.1038/s41467-022-35059-x (PMC9708848; doi:10.1038/s41467-022-35059-x)
Supplement: Supplementary file 3 — Reporting Summary [file 41467_2022_35059_MOESM3_ESM.pdf]

nature portfolio

Corresponding author(s): DD

Last updated by author(s): 09/11/2022

Reporting Summary

Nature Portfolio wishes to improve the reproducibility of the work that we publish. This form provides structure for consistency and transparency in reporting. For further information on Nature Portfolio policies, see our Editorial Policies and the Editorial Policy Checklist.

Statistics

For all statistical analyses, confirm that the following items are present in the figure legend, table legend, main text, or Methods section.

n/a	Confirmed	
☐	☒	The exact sample size (*n*) for each experimental group/condition, given as a discrete number and unit of measurement
☐	☒	A statement on whether measurements were taken from distinct samples or whether the same sample was measured repeatedly
☐	☒	The statistical test(s) used AND whether they are one- or two-sided<br>*Only common tests should be described solely by name; describe more complex techniques in the Methods section.*
☒	☐	A description of all covariates tested
☐	☒	A description of any assumptions or corrections, such as tests of normality and adjustment for multiple comparisons
☐	☒	A full description of the statistical parameters including central tendency (e.g. means) or other basic estimates (e.g. regression coefficient) AND variation (e.g. standard deviation) or associated estimates of uncertainty (e.g. confidence intervals)
☐	☒	For null hypothesis testing, the test statistic (e.g. *F*, *t*, *r*) with confidence intervals, effect sizes, degrees of freedom and *P* value noted<br>*Give P values as exact values whenever suitable.*
☒	☐	For Bayesian analysis, information on the choice of priors and Markov chain Monte Carlo settings
☒	☐	For hierarchical and complex designs, identification of the appropriate level for tests and full reporting of outcomes
☐	☒	Estimates of effect sizes (e.g. Cohen's *d*, Pearson's *r*), indicating how they were calculated

Our web collection on statistics for biologists contains articles on many of the points above.

Software and code

Policy information about availability of computer code

Data collection	UCSC Xena online Browser, Breast Cancer Gene Expression Minor Version V 4.8.
Data analysis	Graph Pad Prism (8.0.1), Image J Heatmapper, Leica Aperio Image Scope Software, Nikon eclipse Ti-S, Ventana Benchmark XT, GSEA 4.2.2 Flow Jo V 10.6.1, FastQC v 0.11.8, MultiQC v 1.8, fastp v 0.20.0, STAR v2, FeatureCounts v 2.0.0, edgeR v 3.28.0, clusterProfiler v 3.14.0, GSEA 4.3.2

For manuscripts utilizing custom algorithms or software that are central to the research but not yet described in published literature, software must be made available to editors and reviewers. We strongly encourage code deposition in a community repository (e.g. GitHub). See the Nature Portfolio guidelines for submitting code & software for further information.

Data

Policy information about availability of data

All manuscripts must include a data availability statement. This statement should provide the following information, where applicable:

- Accession codes, unique identifiers, or web links for publicly available datasets
- A description of any restrictions on data availability
- For clinical datasets or third party data, please ensure that the statement adheres to our policy

Eukaryotic Promoter Database, TRRUST (Version 2), UCSC Xena online Browser (https://ucsc-public-main-xena-hub.s3.us-east-1.amazonaws.com/download/

YauClinical_public%2FYauGeneExp_genomicMatrix.gz; Full metadata) Breast Cancer Gene Expression Minor Version V 4.8 (http://bcgenex.ico.unicancer.fr/BC-GEM/GEM-requete.php), JASPAR (https://jaspar.genereg.net/). The RNA-seq data relevant to this study have been deposited in the Gene Expression Omnibus database under accession code GSE217474. All data needed to evaluate the conclusions in the paper are present in the paper and/or in the Supplementary Information. Additional data related to this manuscript is available in 'Source Data' section.

Human research participants

Policy information about studies involving human research participants and Sex and Gender in Research.

Reporting on sex and gender	The human research participants included in the study were females suffering from Breast Cancer especially TNBC
Population characteristics	The detailed clinical and histological information of the selected 10 subjects included in this study were shown in Supplementary Table S6. Average age (56+/-11.3) years.
Recruitment	A total of 3500 Breast Cancer patients that had reported to RGCIRC between the years 2015-2019. The time frame chosen was such that a 2year follow up period could be accounted for. Out of these 3500 patients , 600 cases qualified as triple negative BC patients based on IHC and Her2 by FISH analysis and who had also undergone surgery and other treatments at RGCIRC. Complete follow-up data of these 600 patients was analyzed to look for disease progression in the form of local recurrence or distant metastasis. Approximately 60 patients had recurred or progressed at varying time points. Most common sites of progression included bone, brain, lung and liver. Of these 60 cases 10 TNBC cases were selected wherein there was a matched tumor block and a matched metastatic site block was available .<br>TNBC cases which were metastatic and could not be included for the study were:<br>1. Cases where the metastatic site was inaccessible for a biopsy and a PET scan imaging suggested towards it being metastatic, a repeat biopsy was not done .<br>2. a large number of cases had depleted blocks, and blocks not containing enough representative tissue for IHC .
Ethics oversight	The study protocol was approved by the Institutional Review Board of the Rajiv Gandhi Cancer Institute and Research Centre (RGCIRC), New Delhi, India (Protocol Number: Res/BR/TRB-24/2021/43) and CSIR-CDRI Institutional Human Ethics Committee (IEC), Lucknow, India (Protocol Number: CDRI/IEC/2022/A9)

Note that full information on the approval of the study protocol must also be provided in the manuscript.

Field-specific reporting

Please select the one below that is the best fit for your research. If you are not sure, read the appropriate sections before making your selection.

☒ Life sciences ☐ Behavioural & social sciences ☐ Ecological, evolutionary & environmental sciences

For a reference copy of the document with all sections, see nature.com/documents/nr-reporting-summary-flat.pdf

Life sciences study design

All studies must disclose on these points even when the disclosure is negative.

Sample size	In vitro experiments were performed in triplicates or otherwise mentioned in the respective Figure legends. The statistical limitations were not taken into account while determining sample size. However, we applied similar experimental circumstances numerous times across biological replicates, resulting in a statistically significant difference between these groups. As a result, no sample size was determined from the outset. For in vivo studies, sample sizes were determined based on our previous experiments. In our experience, n = 5-7 mice per group is sufficient to detect meaningful biological differences with good reproducibility. Treatment/analysis n refers to biologically independent samples as individual mice.
Data exclusions	There were no data that were left out of the analysis.
Replication	The majority of the experiments were carried out numerous times with different biological samples under similar experimental settings, unless otherwise stated in the figure legends, main text, or procedures section.
Randomization	For in vivo experiment, 4-6 week old nude female mice were randomized prior to orthotopic mammary fat pad and subcutaneous inoculation of 4T1 control and experimental cells. No randomization was done for cell culture experiments, same plate of cells were used to set up the treatment groups in each experiment.
Blinding	During the evaluation and collection of data for the IHC quantification experiment, the investigator was blinded. Blinding was not performed for the in vitro and in vivo experiments. For other in-vitro and in-vivo experiments, the investigators needed to know the treatment groups in order to perform the study, and the data analyses were based on objectively measurable data.

Reporting for specific materials, systems and methods

We require information from authors about some types of materials, experimental systems and methods used in many studies. Here, indicate whether each material, system or method listed is relevant to your study. If you are not sure if a list item applies to your research, read the appropriate section before selecting a response.

Materials & experimental systems

n/a	Involved in the study
☐	☒ Antibodies
☐	☒ Eukaryotic cell lines
☒	☐ Palaeontology and archaeology
☐	☒ Animals and other organisms
☒	☐ Clinical data
☒	☐ Dual use research of concern

Methods

n/a	Involved in the study
☒	☐ ChIP-seq
☐	☒ Flow cytometry
☒	☐ MRI-based neuroimaging

Antibodies

Antibodies used	Antibodies for EZH2 (cat# 5246S), H3K27me3 (cat# 9733S), H3K4me3 (cat# 9751S), p-PolII-S5 (cat# 13523S), Sp1 (cat# 9389S) were purchased from Cell Signaling Technology (CST) and used in 1:1000 dilution for WB studies and 1:50 for IHC and ChIP studies wherever applicable. The antibody for GAPDH (#25778, 1:1000) and anti-mouse SP1 (cat# sc-17824, 1:500) were purchased from Santa Cruz Biotechnology. Antibody for KRT14 (cat# ab7800, 1:1000), and Ki-67 (cat# ab16667, 1:1000) were procured from Abcam.
Validation	All antibodies used are commercially available and were tested by the manufacturer. They are standard tools used in the field, and have been previously validated and characterized by multiple laboratories. More information about each antibody and links to product citations can be found on manufacturers' websites. EZH2 (5246S - Cell Signaling Technology, 1:1000) https://www.cellsignal.com/products/primary-antibodies/ezh2-d2c9-xp-rabbit-mab/5246<br>H3k27me3 (cat# 9733S - Cell Signaling Technology, 1:1000): https://www.cellsignal.com/products/primary-antibodies/tri-methyl-histone-h3-lys27-c36b11-rabbit-mab/9733<br>H3k4me3 (cat# 9751S- Cell Signaling Technology, 1:1000): https://www.cellsignal.com/products/primary-antibodies/tri-methyl-histone-h3-lys4-c42d8-rabbit-mab/9751<br>p-PolII-S5 (cat# 13523S- Cell Signaling Technology, 1:1000): https://www.cellsignal.com/products/primary-antibodies/phospho-rpb1-ctd-ser5-d9n5i-rabbit-mab/13523<br>anti-human Sp1 (cat# 9389S- Cell Signaling Technology, 1:1000): https://www.cellsignal.com/products/primary-antibodies/sp1-d4c3-rabbit-mab/9389<br>GAPDH (cat#47724- Cruz Biotechnology, 1:1000): https://www.scbt.com/p/gapdh-antibody-0411<br>anti-mouse SP1 (cat# sc-17824- Cruz Biotechnology, 1:500): https://www.scbt.com/p/sp1-antibody-e-3<br>KRT14 (cat# ab7800 – Abcam, 1:1000): https://www.abcam.com/cytokeratin-14-antibody-ll002-ab7800.html<br>Ki-67 (cat# ab16667 – Abcam, 1:1000): https://www.abcam.com/ki67-antibody-sp6-ab16667.html

Eukaryotic cell lines

Policy information about cell lines and Sex and Gender in Research

Cell line source(s)	HCC-1806, MDA-MB-468, HEK293-T and 4T-1 cell lines were purchased from ATCC, USA.
Authentication	Human cell lines are authenticated by STR profiling from DNA Forensics Laboratory Pvt. Ltd. Vasant Kunj, New Delhi- 110070.
Mycoplasma contamination	All cell lines were tested negative for mycoplasma contamination
Commonly misidentified lines (See ICLAC register)	No commonly misidentified cell lines were used in the study.

Animals and other research organisms

Policy information about studies involving animals; ARRIVE guidelines recommended for reporting animal research, and Sex and Gender in Research

Laboratory animals	4-6 weeks old female nude CD1-Foxn1nu mice were used in this study. Experimental mice were maintained in IVC cages under pathogen-free conditions with a 12h light/12h dark cycle, at 24±2°C temperature with humidity of 45±5%, and were fed with irradiated standard mouse diet at CSIR-CDRI Central Laboratory Animal facility.
Wild animals	This study did not involve wild animals
Reporting on sex	Female mice were only used.
Field-collected samples	No field collected samples are used in this study
Ethics oversight	All animal studies were conducted by following standard principles and procedures approved by the Institutional Animal Ethics Committee (IAEC) of CSIR-Central Drug Research Institute (Protocol Number: IAEC/2018/F-65).

Note that full information on the approval of the study protocol must also be provided in the manuscript.

Flow Cytometry

Plots

Confirm that:

- [x] The axis labels state the marker and fluorochrome used (e.g. CD4-FITC).
- [x] The axis scales are clearly visible. Include numbers along axes only for bottom left plot of group (a 'group' is an analysis of identical markers).
- [x] All plots are contour plots with outliers or pseudocolor plots.
- [x] A numerical value for number of cells or percentage (with statistics) is provided.

Methodology

Sample preparation	Td tomato and GFP positive tumor cells were isolated from primary tumors/metastatic organs and cultured, trypsinized, and evaluated for FL1 and FL2 positivity by flow-cytometry.
Instrument	BD FACSCalibur™ Flow Cytometer
Software	Analyzed by Flow Jo V 10.6.1
Cell population abundance	No cell sorting was done.
Gating strategy	No gating was applied

- [ ] Tick this box to confirm that a figure exemplifying the gating strategy is provided in the Supplementary Information.
